# Development of a novel, urine-based high-risk human papillomavirus polymerase chain reaction test to predict cervical intraepithelial neoplasia abnormalities associated with cervical cancer

**DOI:** 10.1128/spectrum.03796-25

**Published:** 2026-06-17

**Authors:** Vasu Saini, Zhengyang Guo, Jinwen Yu, Di Wu, Hui Du, Peng Yin, Garrett Lee Mosley, Wenkui Dai, Ricky Yin To Chiu

**Affiliations:** 1 Phase Scientific International Ltd., Hong Kong, Hong Kong; 2 Peking University Shenzhen Hospital 74573, Shenzhen, China; London Health Sciences Centre, London, Ontario, Canada

**Keywords:** urine-based HPV testing, self-sampling, cervical cancer screening, cervical intraepithelial neoplasia (CIN) abnormalities, high-risk HPV (hrHPV), qPCR assay, liquid-liquid extraction

## Abstract

This study evaluated and optimized a novel urine-based workflow using high-risk human papillomavirus (hrHPV) polymerase chain reaction (PCR) test to detect 14 hrHPV genotypes linked to cervical intraepithelial neoplasia (CIN) abnormalities for cervical cancer screening. In cohort 1 for clinical feasibility, 82 women with confirmed CIN2+ abnormalities provided paired urine and cervical brush samples before treatment. Urine was tested with the novel hrHPV workflow, and cervical brushes with the Cobas HPV Test (Roche). For Ct cut-off optimization in cohort 2, urine samples from 983 women in a primary screening, triage, and follow-up program were analyzed, optimizing CIN2+ and CIN3+ sensitivity and specificity by varying the 12-other hrHPV channel Ct cut-off from 28.0 to 38.0, while maintaining HPV16/18 Ct cut-offs at 38.0. In cohort 1, the urine hrHPV test showed 92.7% sensitivity for CIN2+ and 93.9% for CIN3+, exceeding cervical brush sensitivities of 78.1% and 90.9%, respectively. In cohort 2, the 12-other hrHPV Ct altered CIN2+ sensitivity from 76.6% to 93.6% and specificity from 83.1% to 75%. ROC analysis identified optimal cut-offs at 32.76 for CIN2+ and 30.47 for CIN3+. The proposed cut-offs are HPV16/18 at 38.0, and 12 other hrHPV at 33.0 for maximum sensitivity (93.62% CIN2+, 90.91% CIN3+) or at 30.5 for balanced performance (89.36% CIN2+ sensitivity, 80% specificity). The urine-based hrHPV test uses 40 mL of urine collected using a standard urine collection device, and demonstrated high sensitivity for CIN2+ prediction. Adjustable Ct thresholds allow tailored performance optimization, thereby enhancing accessibility, adherence, and early cervical cancer detection across diverse clinical settings.

**IMPORTANCE:**

A novel urine-based high-risk human papillomavirus (hrHPV) polymerase chain reaction test was developed to detect 14 hrHPV genotypes, including individual HPV16 and HPV18 typing, associated with cervical cancer progression. By employing a liquid-liquid separation method to extract DNA from large-volume (40 mL) pot-collected urine samples, the test demonstrated sensitivities of 92.7% for cervical intraepithelial neoplasia grade 2 or higher (CIN2+) and 93.9% for CIN3+. Optimization of cycle threshold cut-offs yielded 93% sensitivity and 77% specificity for predicting CIN2+ in an enriched population. It can enhance accessibility, compliance, and early detection across diverse clinical settings.

## INTRODUCTION

Over 99.7% of cervical cancer cases are linked to persistent high-risk human papillomavirus (hrHPV) infection, with high-risk types 16 and 18 responsible for approximately 70% of cases (1, 2). In addition to vaccination, routine screening to identify and quickly treat pre-cancerous lesions, typically through cryotherapy or thermal ablation, is an effective approach for managing disease progression within a population. According to the World Health Organization, HPV DNA-based screening is a more effective strategy than traditional cytology-based methods, such as Pap smear, due to its ability to detect the oncovirus prior to cellular changes, facilitating earlier intervention, especially in resource-limited settings (3).

While many countries promote routine HPV screening within their healthcare systems, there are still significant barriers to consistent implementation and patient adherence, largely due to inconvenience, fear of pain during the procedure, psychosocial factors, such as embarrassment and perceived discomfort associated with the pelvic examination, cultural beliefs and stigmas surrounding gynecological health and sexually transmitted infections, limited access to healthcare facilities, or procrastination to visit the healthcare facility for cervical sampling. As of 2023, about 75% of women aged 21–65 years in the United States were up to date with cervical cancer screening. In contrast, only about half of women aged 35–64 years in China had undergone cervical cancer screening in their lifetime (4, 5). Self-collected samples, including vaginal swabs and urine samples, offer a convenient and typically more cost-effective alternative to traditional clinician-collected sampling methods for cervical cancer screening (6, 7). Additionally, relative to cervical and vaginal sampling, self-collected urine is non-invasive, painless, and can be conveniently collected from the privacy of the home or in a healthcare setting. The option to self-collect samples at home and mail them to health clinics for analysis can improve screening rates, especially among populations with low participation due to logistical challenges, limited healthcare resources, or cultural stigma.

While urine-based testing is a promising approach, historically it has struggled to consistently demonstrate the sufficient sensitivity required to be a viable sample type for primary cervical cancer screening (8–10). For example, a cross-sectional study published in the Asian Pacific Journal of Cancer Prevention in 2019, involving 114 cervical cancer cases, found HPV DNA positivity in 48.2% of urine samples compared to 78.1% in cervical samples, with a sensitivity of 59.6% and specificity of 92% (11). This is likely due to the diluted concentration of HPV DNA in urine samples relative to vaginal or cervical sampling, as well as the volume processing limitations of current urine extraction kits. More recently, several studies have demonstrated that hrHPV testing can achieve comparable clinical accuracy to cervical sample testing by using a small volume of first-void urine samples (12–15). The VALHUDES studies have focused on evaluating first-void urine samples due to the volume-processing limitations of current extraction kits and to make use of pre-existing infrastructure for screening tests, including automated instruments like cobas 4800 (Roche Molecular Systems, Pleasanton, CA) and Alinity (Abbott, USA) platforms. However, such specimen restrictions can be problematic, as demonstrated by Sabeena et al., who found that in order to achieve high sensitivity, first-void urine collection necessitates greater standardization or the adoption of specialized collection devices, a finding corroborated by VALHUDES research.

It is well known that the detection of HPV does not perfectly correlate with the presence or development of pre-cancerous lesions due to transient HPV infections that are naturally cleared by the immune system. Consequently, it is important to have an optimal balance between an assay’s clinical sensitivity and specificity, depending on the intended use in a cervical cancer screening program. Tests overly sensitive for the presence of HPV DNA may lead to excessive burden on the healthcare system due to unnecessary follow-up examinations, while insufficient sensitivity for the viral detection may result in missed pre-cancerous lesions and delayed intervention. Moreover, there is ongoing debate within scientific and medical groups about how to treat cervical intraepithelial neoplasia grade 2 (CIN2) pre-cancerous lesions, particularly in younger women (16, 17). This lack of consensus is further nuanced by differences in screening and treatment programs between regions with varying access to healthcare resources and reflects broader challenges about balancing early detection, the costs of overtreatment, and diagnostic accuracy. Therefore, the choice of screening strategy must consider country demographics, public health priorities, and healthcare facilities while accounting for the trade-off between the clinical sensitivity and specificity of a test.

In this study, we evaluated a novel urine-based qPCR hrHPV test (PHASE HPV Urine Test, PHASE Scientific, HK) that utilizes the PHASiFY Max Total DNA Extraction kit to detect 14 genotypes of hrHPV DNA with individual subtype detection of HPV 16 and HPV 18. By leveraging the unique liquid-liquid phase separation technology capable of processing 40 mL of urine, coupled with a semi-automated magnetic bead-based DNA extraction method, the concentration of extracted viral DNA was increased without restricting specimens to first-void urine (18). First, to demonstrate clinical feasibility, we evaluated the performance of the assay in 82 confirmed CIN2+ positive patients and compared the results with clinician-collected cervical samples tested with Roche cobas 4800 HPV Test (Roche Molecular Systems, Pleasanton, CA). Second, to demonstrate the viability of the test within differing cervical cancer screening programs, we evaluated the impact of adjusting the cycle threshold (Ct) cut-off values on the balance of clinical sensitivity and specificity by testing 983 participants in a combined screening and enriched cohort.

## MATERIALS AND METHODS

### Study participants and sample collection

This study was designed to determine the optimal clinical Ct cut-off values for the PHASE HPV Urine test to identify women at risk of having CIN2+ from women with <CIN2 pathology. The study was conducted with the data from the Self-Collected Cervical Cancer Prevention Study. Non-pregnant women aged 30–64 years with no history of radical trachelectomy were enrolled with informed consent at Peking University Shenzhen Hospital (PUSH), Baise People’s Hospital, Baise Women’s and Children’s Hospital, and Tiandong People’s Hospital for this study. Enrolled participants were distributed into two different cohorts. Cohort 1 comprised women who were diagnosed with CIN2 or higher lesions following colposcopy examination and subsequent histopathological evaluation of their tissue biopsies. All participants subsequently returned for treatment, receiving either a loop electrosurgical excision procedure (LEEP) or thermal ablation. On the day of their scheduled surgical procedure, a paired physician-collected cervical brush specimen and a 40–100 mL urine sample were collected from each participant for further analysis prior to their surgical procedure. Cohort 2 consisted of women who registered for a nationwide primary hrHPV screening program (primary screening cases), women who tested positive for hrHPV DNA during the primary screening program and returned for triage management (triage cases), and women who either returned for the first-year follow-up due to a positive result from the primary HPV screening test or received treatment with thermal ablation or LEEP and returned for first-year follow-up (first-year follow-up cases). All the participants provided 40–100 mL of urine on the day of their hospital visit.

For a physician-collected cervical smear sample, a trained physician collected a cervical sample for each participant using a conical brush after inserting the speculum into the vagina. The collected cervical smear samples were resuspended in 10–15 mL of ThinPrep PreservCyt resuspension medium (Hologic, Marlborough, MA). The preserved cervical smear samples were sent to the PUSH lab for further testing. One milliliter of each cervical smear sample was tested using the gold standard FDA-approved Roche cobas 4800 HPV Test. In parallel, each participant collected 40–100 mL of urine using the INDICAID Urine Collection Kit (Phase Scientific International Ltd., Hong Kong). Each INDICAID Urine Collection Kit consists of a labeled 120 mL bottle containing ~5 mL of a DNA preservative solution capable of inhibiting the action of DNases and a urine collection cup. After collection, the urine samples were sent to Phase Scientific Independent Clinical Laboratory in Shenzhen, China, for further processing. All the urine samples were stored at 4°C until processing.

### DNA extraction from urine

For urine samples with added DNA preservative solution, a semi-automated workflow of the PHASiFY MAX Total DNA Extraction Kit was used to extract urinary DNA from 40 mL of urine. Briefly, 40 mL of urine was incubated at 37°C for 15 min for lysis. The lysate was subjected to two sequential liquid-liquid purification and concentration steps. The lysate was added to the first liquid-liquid phase-forming solution and vortexed to form a homogeneous mixture. The resulting homogeneous solution was centrifuged at 2,300 × *g* for 6 min to facilitate liquid-liquid phase separation. Following the phase separation, the bottom phase was extracted and transferred to the second liquid-liquid phase-forming solution. The resulting solution was vortexed and centrifuged at 2,300 × *g* for 6 min to allow liquid-liquid phase separation. The resulting top phase (~600 µL) containing concentrated DNA was then processed by magnetic bead-based purification using the Tianlong GeneRotex 48 instrument (an automatic nucleic acid extractor from Xi’an Tianlong Science and Technology Co., Ltd. (Tianlong, Shaanxi, China). The DNA was eluted in the 60 µL MilliQ water.

### High-risk HPV PCR test kit

The High-risk HPV PCR kit is a qualitative *in vitro* test designed to detect HPV DNA in human urine samples (Phase Scientific International Ltd., Hong Kong). The qPCR assay amplifies and detects 14 hrHPV subtypes in a single PCR reaction. The assay includes testing of 14 hrHPV genotypes, including 16, 18, 31, 33, 35, 39, 45, 51, 52, 56, 58, 59, 66, and 68, and distinguishes HPV16 and HPV18 from 12 other hrHPV types (Table 1). The PCR kit utilizes human β-globin as an internal control for quality assurance. The HPV urine test was performed according to the manufacturer’s instructions (Table 2).

**TABLE 1:** Probe description for detection channels

Detector name	Target	Reporter dye	Quencher
FAM	12-other hrHPV types (31, 33, 35, 39, 45, 51, 52, 56, 58, 59, 66, 68)	FAM	None
CY5	HPV16	None	None
ROX	HPV18	ROX	None
VIC	Internal control	VIC	None

**TABLE 2:** PCR cycle

Step	No. of cycles	Temperature (°C)	Time	Fluorescence detection
1	1	37	2 min	/^*a*^
2	1	95	3 min	/
3	45	95	10 s	/
4		60	35 s	FAM, VIC, ROX, CY5

^*a*^ “/” indicates that no fluorescence was detected during those steps.

The samples were run on the QuantStudio 5 instrument, and the Ct values were exported from the QuantStudio Design & Analysis Software v1.5.3.

### PHASE HPV urine test

The PHASE HPV urine test is a qualitative, real-time PCR test designed to detect 14 hrHPV subtypes with individual typing of HPV 16 and HPV 18, in DNA obtained from urine. The test is performed using three kits for (i) urine sample collection (INDICAID Urine Collection Kit), (ii) urine DNA extraction (PHASiFY Max Total DNA Extraction kit), and (iii) hrHPV qPCR Assay (High-Risk HPV PCR Test Kit) using urine DNA on the QuantStudio 5 platform (Thermo Fisher Scientific Inc., USA).

### Data analysis

#### Ct cut-off analysis for the 12-other hrHPV channels

The Ct cut-off values were set at 38.0 for the HPV16 and HPV18 detection channels. For the channel detecting the 12 other hrHPV subtypes, the Ct cut-off value was varied from 28.0 to 38.0 in increments of 0.5. At each increment, the overall clinical sensitivity and specificity of the PHASE HPV Urine test were calculated. Sensitivity was determined by considering CIN2+ cases as positives and <CIN2 cases as negatives for the CIN2+ classification, and CIN3+ cases as positives and <CIN3 cases as negatives for the CIN3+ classification.

#### Receiver-operating-characteristic curve and precision-recall analysis

The HPV16 and/or HPV18 positive cases were excluded from the 983 sample pool for the receiver-operating-characteristic (ROC) curve and precision-recall area under the curve (PR-AUC) analysis in order to evaluate the impact of varying the Ct cut-off value for the 12-other hrHPV. Out of a total of 983 samples, 935 samples that were negative for HPV16 and/or HPV18 subtypes were used to evaluate the Ct cut-off for the CIN2+ and CIN3+ classification. A linear regression analysis was performed to evaluate the Ct cut-off for the CIN2+ and CIN3+ based classification. The regression analysis was performed by considering CIN2+ cases as positives and <CIN2 cases as negatives for the CIN2+ classification, and CIN3+ cases as positives and <CIN3 cases as negatives for the CIN3+ classification.

Similarly, the ROC curve and PR-AUC analysis for HPV16 and HPV18 channels were performed using all 983 cases. ROCs and PR-AUCs were analyzed by Medcalc software (MedCalc Software Ltd., Belgium).

## RESULTS

### Clinical feasibility evaluation in confirmed CIN2+ cohort

**Fig 1:**
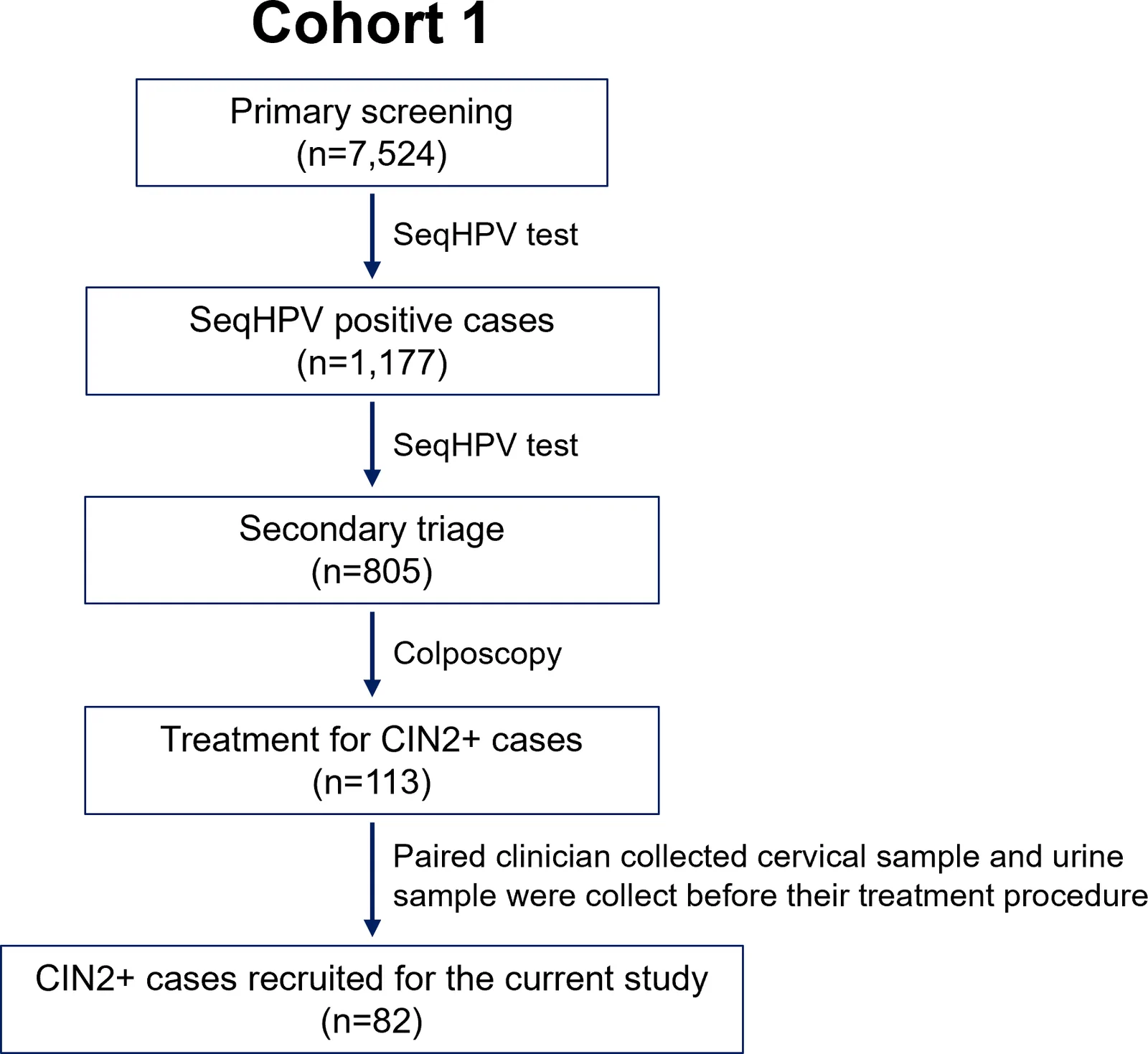
Program workflow and sample collection sequence for the female participants included in cohort 1.

Figure 1 demonstrates the flow of participant recruitment and study inclusion. Women with confirmed CIN2+ diagnoses were recruited to provide self-collected urine samples and paired clinician-collected cervical samples prior to the treatment procedure.

The hrHPV urine test demonstrated a sensitivity of 92.7% to detect CIN2+ cases, and 93.9% to detect CIN3+ cases (Table 3). In contrast, the cobas 4800 HPV Test showed a sensitivity of 78.1% for CIN2+ cases and 90.9% for CIN3+ cases.

**TABLE 3:** Sensitivity for CIN2+ and CIN3+ cases with the HPV urine test from cohort 1

PHASE-hrHPV Ct cut-off value	PHASE-hrHPV		Cobas 4800 (as per IFU’s instructions)	
	Colposcopy results CIN2+	Colposcopy results CIN3+	Colposcopy results CIN2+	Colposcopy results CIN3+
	Point estimate 95% CI	Point estimate 95% CI	Point estimate 95% CI	Point estimate 95% CI
HPV16: 45.0	17.07 (14/82) (9.66–26.98)	18.18 (6/33) (6.98–35.46)	13.41 (11/82) (6.89–22.74)	15.15 (5/33) (5.11–31.90)
HPV18: 45.0	6.10 (5/82) (2.01–13.66)	9.09 (3/33) (1.92–24.33)	6.10 (5/82) (2.01–13.66)	9.09 (3/33) (1.92–24.33)
HPV12+: 45.0	75.61 (62/82) (64.88–84.42)	78.79 (26/33) (61.09–91.02)	60.98 (50/82) (49.57–71.56)	69.70 (23/33) (51.29–84.41)
HPV16: 45.0, HPV18: 45.0, HPV12+: 45.0	92.68 (76/82) (84.75–97.27)	93.93 (31/33) (79.77–99.26)	78.05 (64/82) (67.55–86.44)	90.91 (30/33) (75.67–98.09)

### qPCR cycle threshold cut-off value impact on screening and enriched cohort

**Fig 2:**
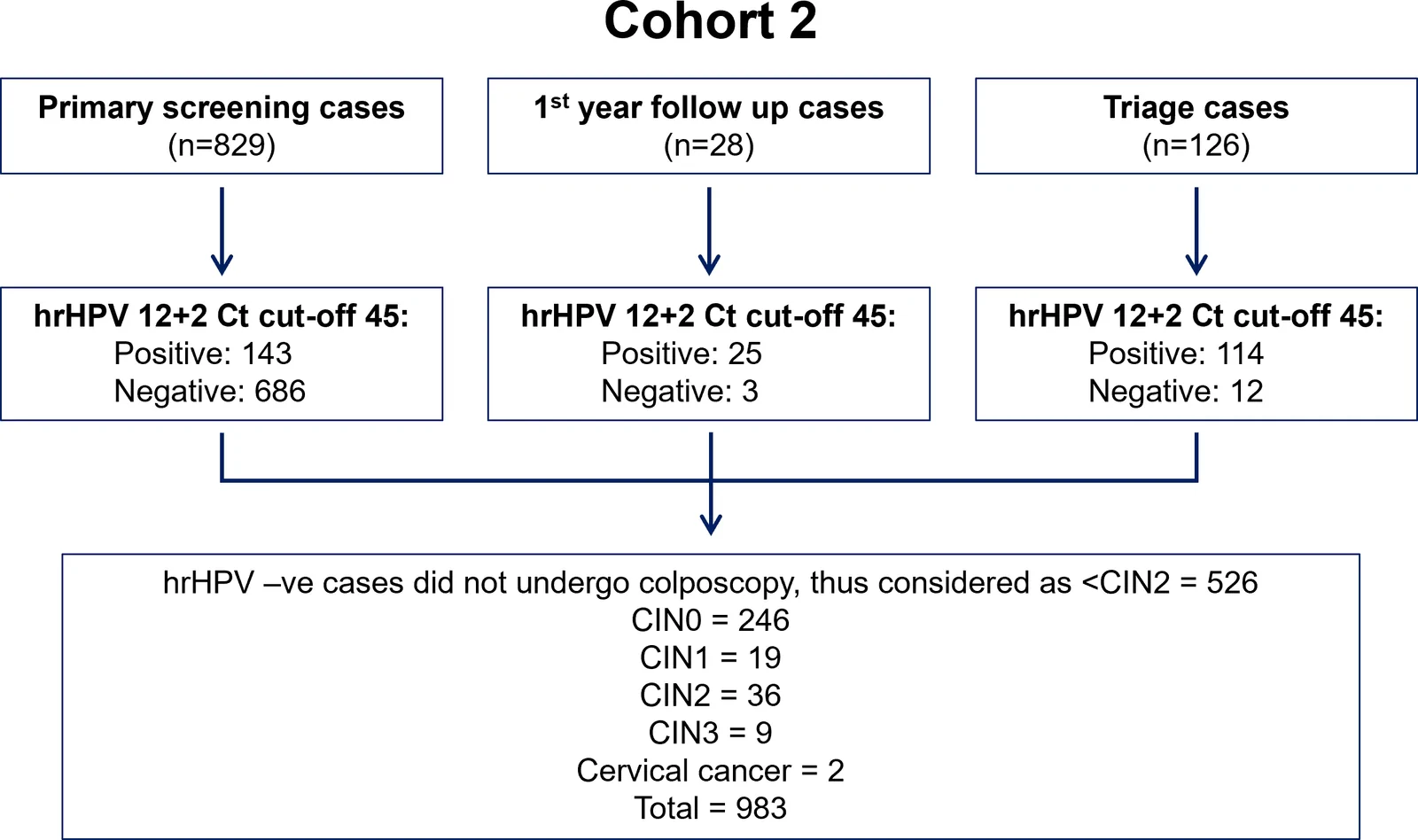
Flowchart detailing the participants included in cohort 2 and their histopathological distribution.

After demonstrating that the urine-based assay had sufficient clinical sensitivity in identifying known positive CIN2+ patients, the impact of adjusting the qPCR Ct cut-off value on the balance of clinical sensitivity and specificity was evaluated on a larger cohort. To do this, individuals enrolled in a broad cervical cancer screening program were recruited to provide samples for analysis. A total of 983 urine samples were obtained from women who were appearing for primary screening (*n* = 829), an enriched triage population (*n* = 126), and first year follow-up women who tested positive during their initial hrHPV testing round (*n* = 28) with SeqHPV (a multiplex PCR based NGS assay to detect all 14 hrHPV subtypes, i.e., HPV-16, -18, -31, -33, -35, -39, -45, -51, -52, -56, -58, -59, -66, and -68 with individual genotyping, developed by BGI-Shenzhen, China) (Fig. 2). hrHPV negative samples from patients who did not undergo follow-up colposcopy procedures were assumed to be <CIN2 cases. Of 983 participants, 36, 9, and 2 patients were positive for CIN2, CIN3, and cervical cancer, respectively.

**Fig 3:**
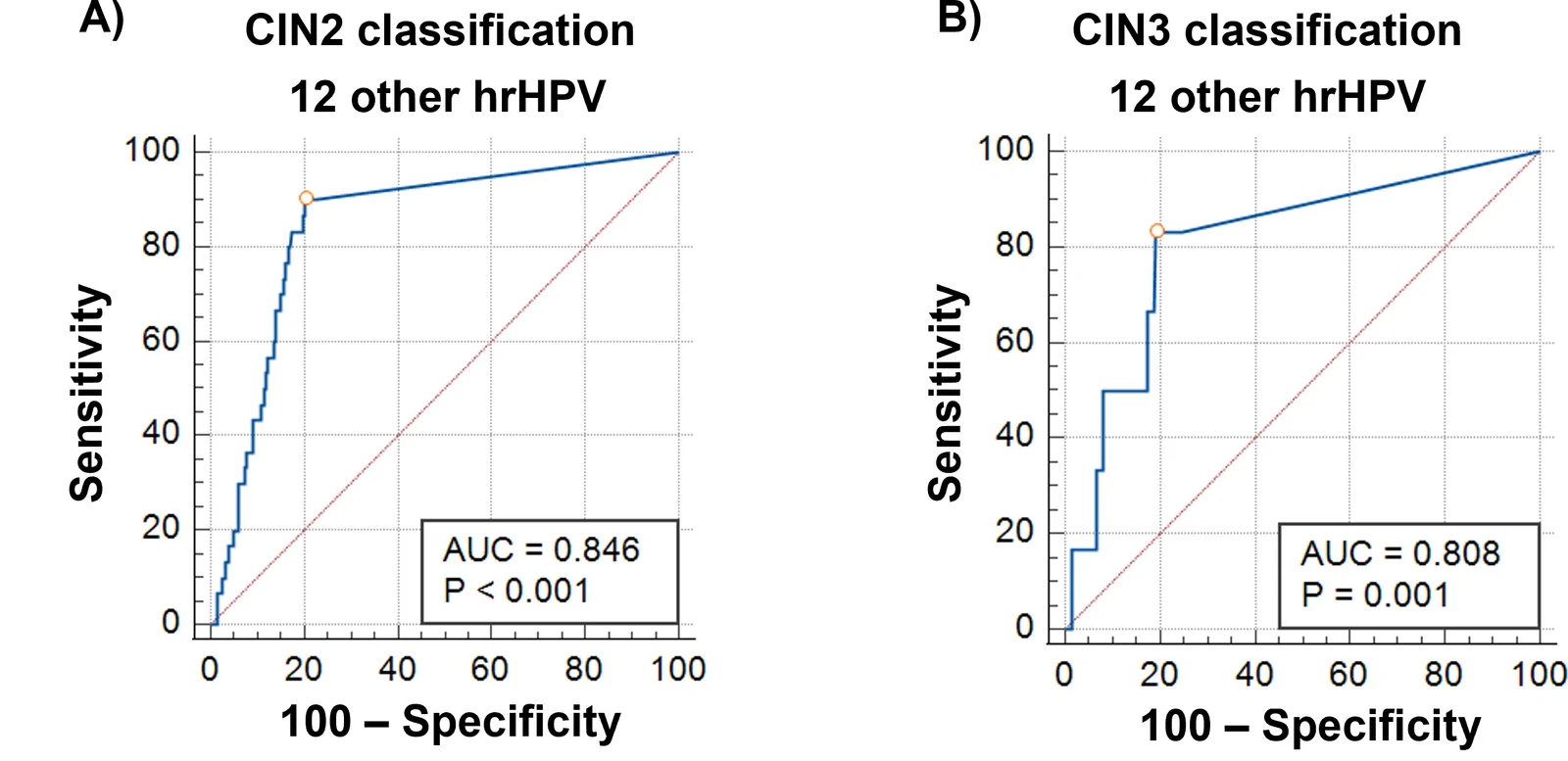
ROC curve analysis for evaluating the impact of varying the Ct cut-off values for the 12 other hrHPV channels to distinguish (**A**) CIN2+ from <CIN2, and (**B**) CIN3+ from <CIN3.

While evaluating the Ct cut-off of the 12 other hrHPV channels, the HPV16 and/or HPV18 positive cases were excluded from the 983 sample pool to negate the risk factor associated with HPV16 and/or HPV18 infection. ROC curve analysis was performed on a subset of 935 samples, which were either 12 other hrHPV positive and 12 other hrHPV negative. Two classification approaches were used: (i) distinguishing CIN2+ from <CIN2, and (ii) distinguishing CIN3+ from <CIN3. ROC curve analysis determined the Ct cut-offs to be 32.76 for CIN2+ cases and 30.47 for CIN3+ cases (Fig. 3). The ROC curve analysis of HPV16 and HPV18 determined the Ct cut-offs to be 33.8 and 35.57, respectively (Fig. S1). However, given the high carcinogenicity of HPV16 and 18 subtypes, the Ct cut-off values were fixed at 38.0 to maximize the detection of patients with HPV16 and 18 infections.

The overall sensitivity and specificity of the HPV urine test were then evaluated by fixing the Ct cut-off values of HPV16 and HPV18 Ct and varying the 12 other hrHPV channel cut-off values from 28.0 to 38.0 in increments of 0.5. Table 4 shows that as the Ct cut-off value increased, the sensitivity for CIN2+ classification increased from 76.6% and plateaued at 93.6%, while the specificity decreased from 83.1% to 75.4%. Similarly, the sensitivity for CIN3+ classification increased from 72.7% and plateaued at 90.9%, while the specificity decreased from 80.9% to 72.8%.

**TABLE 4:** List of sensitivity and specificity values for CIN2+ and CIN3+ classification in response to varying the Ct cut-off value for the 12 other hrHPV channels from 28.0 to 38.0^*a*^

	PHASE-hrHPV test			
PHASE-hrHPV Ct cut-off value	CIN2+ classification		CIN3+ classification	
	Sensitivity (%)	Specificity (%)	Sensitivity (%)	Specificity (%)
HPV16: 38.0, HPV18: 38.0, HPV12+: 28.0	76.60 (36/47)	83.12 (778/936)	72.73 (8/11)	80.86 (786/972)
HPV16: 38.0, HPV18: 38.0, HPV12+: 28.5	78.72 (37/47)	82.59 (773/936)	72.73 (8/11)	80.23 (780/972)
HPV16: 38.0, HPV18: 38.0, HPV12+: 29.0	80.85 (38/47)	81.52 (763/936)	72.73 (8/11)	79.12 (769/972)
HPV16: 38.0, HPV18: 38.0, HPV12+: 29.5	85.11 (40/47)	80.66 (755/936)	81.82 (9/11)	78.19 (760/972)
HPV16: 38.0, HPV18: 38.0, HPV12+: 30.0	87.23 (41/47)	80.24 (751/936)	81.82 (9/11)	77.68 (755/972)
HPV16: 38.0, HPV18: 38.0, HPV12+: 30.5	89.36 (42/47)	79.92 (748/936)	90.91 (10/11)	77.37 (752/972)
HPV16: 38.0, HPV18: 38.0, HPV12+: 31.0	89.36 (42/47)	78.74 (737/936)	90.91 (10/11)	76.24 (741/972)
HPV16: 38.0, HPV18: 38.0, HPV12+: 31.5	89.36 (42/47)	78.31 (733/936)	90.91 (10/11)	75.82 (737/972)
HPV16: 38.0, HPV18: 38.0, HPV12+: 32.0	89.36 (42/47)	77.89 (729/936)	90.91 (10/11)	75.41 (733/972)
HPV16: 38.0, HPV18: 38.0, HPV12+: 32.5	91.49 (43/47)	77.24 (723/936)	90.91 (10/11)	74.69 (726/972)
HPV16: 38.0, HPV18: 38.0, HPV12+: 33.0	93.62 (44/47)	77.03 (721/936)	90.91 (10/11)	74.38 (723/972)
HPV16: 38.0, HPV18: 38.0, HPV12+: 33.5	93.62 (44/47)	76.71 (718/936)	90.91 (10/11)	74.07 (720/972)
HPV16: 38.0, HPV18: 38.0, HPV12+: 34.0	93.62 (44/47)	76.18 (713/936)	90.91 (10/11)	73.56 (715/972)
HPV16: 38.0, HPV18: 38.0, HPV12+: 34.5	93.62 (44/47)	75.75 (709/936)	90.91 (10/11)	73.15 (711/972)
HPV16: 38.0, HPV18: 38.0, HPV12+: 35.0	93.62 (44/47)	75.75 (709/936)	90.91 (10/11)	73.15 (711/972)
HPV16: 38.0, HPV18: 38.0, HPV12+: 35.5	93.62 (44/47)	75.64 (708/936)	90.91 (10/11)	73.05 (710/972)
HPV16: 38.0, HPV18: 38.0, HPV12+: 36.0	93.62 (44/47)	75.64 (708/936)	90.91 (10/11)	73.05 (710/972)
HPV16: 38.0, HPV18: 38.0, HPV12+: 36.5	93.62 (44/47)	75.43 (706/936)	90.91 (10/11)	72.84 (708/972)
HPV16: 38.0, HPV18: 38.0, HPV12+: 37.0	93.62 (44/47)	75.43 (706/936)	90.91 (10/11)	72.84 (708/972)
HPV16: 38.0, HPV18: 38.0, HPV12+: 37.5	93.62 (44/47)	75.43 (706/936)	90.91 (10/11)	72.84 (708/972)
HPV16: 38.0, HPV18: 38.0, HPV12+: 38.0	93.62 (44/47)	75.43 (706/936)	90.91 (10/11)	72.84 (708/972)

^*a*^ The Ct value cut-off of HPV16 and 18 was held constant at 38.0.

Finally, we evaluated the genotype distribution of HPV16, HPV18, and the 12 other hrHPV types among <CIN2 and CIN2+ cases (Table 5). Among women with CIN2+ lesions, the positivity rates were 27.66% for HPV16 and 8.51% for HPV18, compared with 68.08%–76.59% for the 12 other hrHPV types. In contrast, the positivity for HPV16 (1.71%) and HPV18 (1.60%) was markedly lower than for the 12 other hrHPV types (18.38%–21.69%).

**TABLE 5:** Genotype distribution of hrHPV subtypes at different selected Ct cut-offs for <CIN2 and CIN2+ population

hrHPV subtype channels and cut-offs	For <CIN2 cases (*n* = 936)	For CIN2+ cases (*n* = 47)
HPV16 (Ct cut-off: 38.0)	1.71% (16/936)	27.66% (13/47)
HPV18 (Ct cut-off: 38.0)	1.60% (15/936)	8.51% (4/47)
12 other hrHPV (Ct cut-off: 33.0)	21.69% (203/936)	76.59% (36/47)
12 other hrHPV (Ct cut-off: 30.5)	18.38% (172/936)	68.08% (32/47)
hrHPV 12+2 (Ct cut-off A)	22.97% (215/936)	93.62% (44/47)
hrHPV 12+2 (Ct cut-off B)	20.09% (188/936)	89.36% (42/47)

## DISCUSSION

We completed a study of a novel urine-based hrHPV detection assay. The assay performance data from the 82 confirmed CIN2+ patients (Table 3) demonstrate the clinical feasibility of using self-collected urine samples for primary screening of cervical cancer without the need for isolating first void urine from the rest of the urine stream. More specifically, the assay achieved a sufficiently high sensitivity of 92.7% and 93.9% for CIN2+ and CIN3+ identification, respectively. This was directly compared to paired clinician-collected cervical samples run on the cobas 4800 HPV Test. While the cobas 4800 HPV Test is an FDA-approved assay optimized for use with 20 mL of ThinPrep PreservCyt medium and employs a Ct cut-off value calibrated to balance sensitivity and specificity for primary cervical cancer screening, clinician-collected cervical samples in this study were resuspended in only 10–15 mL of the medium. This protocol deviation may have contributed to the observed reduction in sensitivity for the Roche assay, which deviated from its established clinical performance benchmarks. It is important to recognize that the Cobas HPV Test has an optimized Ct cut-off value to balance the sensitivity and specificity of the assay for primary cervical cancer screening, while the Ct cut-off value for the urine assay was intentionally set at a maximum of 45 for HPV types 16, 18, and 12+ for this initial clinical evaluation. While the sensitivity for detecting CIN2+ cases in the enriched cohort 1 is high for the novel urine-based hrHPV test, these values are not intended to represent the assay’s final clinical performance. Instead, they demonstrated that, unlike previous attempts at developing a urine-based primary screening assay, the “ceiling” sensitivity was sufficiently high enough to subsequently optimize the Ct cut-off value to balance the sensitivity and specificity of the assay.

We believe the high sensitivity was enabled by the unique liquid-liquid extraction system which processes significantly larger volumes of urine (40 mL) and as a result, captures more of the HPV DNA without the need for isolating first void urine. The collection of first void urine involves capturing a select portion of the urine which has the highest relative concentration of the viral DNA and minimizing dilution from mixing with the lower concentration portions of the urine stream. There are inherent limitations to this approach, as each patient’s DNA concentration profile will differ and only a limited amount of urine volume can be processed. As discussed previously, standardization of first void collection devices is also an operational and regulatory challenge. It should be noted that the large urine volume liquid-liquid extraction technique used in this study and first void collections are not mutually exclusive approaches to improving urine HPV assay sensitivity and may benefit from working in tandem.

With any HPV assay used for primary screening of cervical cancer, there is a concern for overcalling hrHPV cases and thus overburdening the healthcare system with unnecessary follow-up examinations. To address this risk for the PHASE HPV Urine Test, we evaluated the impact of changing the Ct cut-off value when testing 983 participants from a combined primary screening and enriched cohort. Considering the higher rate of high-grade neoplasia progression associated with HPV16 and HPV18 infection, a Ct cut-off of 38.0 was held constant for both HPV16 and HPV18 channels. For the 12-other hrHPV subtypes, as the Ct cut-off value increased, we observed an expected inverse relationship with an increasing sensitivity and decreasing specificity for identification of CIN2+ and CIN3+ (Table 4). It is also noted that the sensitivities at the Ct cut-off 38 for all channels were 93.6% and 90.9% for CIN2+ and CIN3+, respectively, which are similar to the values in the initial evaluation of the 82 confirmed CIN2+ patients from the first cohort.

It is important to note that what is considered the optimal Ct cut-off value may vary depending on the available healthcare resources within a region or on the objectives of a cervical cancer screening and treatment program. The decision of whether or not to treat a CIN2 pre-cancerous lesion is still under active discussion by policymakers and is dependent on screening and treatment strategy; therefore, we identified two Ct cut-off values that may be of distinct value: (i) HPV16: Ct 38.0, HPV18: Ct 38.0, and 12 other hrHPV: Ct 33.0, which represents the lowest cut-off that achieves the maximum plateau sensitivity for CIN2+ and CIN3+, and (ii) HPV16: Ct 38.0, HPV18: Ct 38.0, and 12 other hrHPV: Ct 30.5, which maximizes the CIN2+ sensitivity while maintaining a higher CIN2+ specificity (Table 4; Fig. 3). By way of example, cut-off A may be more suitable for programs that prioritize disease identification and minimizing missed pre-cancerous lesions, while cut-off B may be more suitable for programs that have to prioritize management of limited healthcare resources and want to mitigate unnecessary follow-up evaluations.

It is important to note that the detection of hrHPV DNA indicates the presence of viral infection but does not necessarily equate to the existence of CIN2+ lesions. As the majority of HPV infections are transient and are spontaneously cleared by the immune system, and progression to high-grade neoplasia typically requires persistent infection, many hrHPV-positive women in a screening population may not harbor underlying CIN2+ at the time of testing. This fundamental characteristic of the biology of HPV infection inherently constrains the maximum achievable PR-AUC for any standalone HPV-based test and thereby accounts for the modest values observed in the present study (Fig. S2). These observations align with the previously reported performance metrics reported in a large Chinese screening cohort, where the positive predictive value (PPV) and sensitivity points for the Cobas 4800 HPV test (sensitivity: 82.14, PPV: 12.64) and the HC2 test (sensitivity: 92.86, PPV: 8.87) were comparable to the values for PHASE HPV Urine Test (sensitivity: 93.62, PPV: 16.99) discussed in this study (19).

Notably, the participation rate of younger women was higher than that of the older women (Table S1). Moreover, the prevalence of hrHPV-positive cases was higher in younger women compared to the older population (Table S1), a finding which is similar to previous studies (20, 21). The positivity rates observed for HPV16 and HPV18 were relatively lower compared to the 12 other hrHPV types in this cohort and are consistent with previous reports (Table 5) (22), and likely reflect the higher number of concurrent infections captured in the pooled 12 other hrHPV channel, as well as regional differences in genotype prevalence. Importantly, however, the progression rate to CIN2+ was markedly higher among HPV16 and HPV18 positive women, underscoring the well-established high oncogenic potential of these two genotypes and the need to maximize the detection of HPV16 and HPV18 cases (Table S2) (2, 22, 23). While for the remaining 12 hrHPV subtypes, cervical cytology, DNA methylation, HPV methylation, or continued observation and repeat testing to identify persistent infections can be used as a triage strategy for patient management.

In conclusion, our findings demonstrate the clinical feasibility of using urine-based HPV testing for cervical cancer screening without the need for the isolation of first void urine. However, this study serves as a feasibility demonstration of urine-based testing and should not be considered as establishing final performance metrics. For instance, a primary limitation is the classification of hrHPV-negative cases as <CIN2 in the screening population, as these cases lacked verification through comparator tests and were not followed up with colposcopy, rendering cytology or colposcopy results unavailable. Consequently, this approach may have misclassified false-negative cases as <CIN2, thereby inflating specificity estimates. Additional limitations include the use of an enriched cohort and the relatively small sample size compared to the recommended large prospective cohort for validation of assays intended for use as large population screening tests. Furthermore, despite using all the 983 cases due to the limited number of HPV16 and HPV18 positive cases, it was challenging to conduct a robust regression analysis (Fig. S1). Thus, a constant Ct cut-off of 38.0 was selected to maximize the HPV16 and HPV18 detection during this initial testing. This approach enabled the determination of a suitable Ct cut-off value, which helped minimize false positive cases during the detection of HPV16 and HPV18. To overcome these limitations, follow-up large-scale studies are underway to evaluate performance in a large prospective screening population with a broad representation of relevant demographics. To our knowledge, this is the first example of a urine-based HPV assay that demonstrates sufficient sensitivity to enable tailoring of Ct cut-off values and subsequent balancing of sensitivity and specificity to meet specific cervical screening program requirements. We believe our unique results were enabled by the novel liquid-liquid extraction system that can process significantly larger volumes of urine than traditional extraction kits. The potential impact of a reliable urine-based cervical cancer screening assay should not be understated. The ability to improve testing accessibility, increase patient adherence, and reduce healthcare costs is significant and merits further investigation.

## SUPPLEMENTAL MATERIAL

Supplemental material spectrum.03796-25-s0001.docx Fig. S1 and S2; Tables S1 and S2.

ASM does not own the copyrights to Supplemental Material that may be linked to, or accessed through, an article. The authors have granted ASM a non-exclusive, world-wide license to publish the Supplemental Material files. Please contact the corresponding author directly for reuse.
