# Supplementary material for: Development of a novel, urine-based high-risk human papillomavirus polymerase chain reaction test to predict cervical intraepithelial neoplasia abnormalities associated with cervical cancer
Source: Microbiol Spectr. 2026 Jun 17;14(7):e03796-25. doi: 10.1128/spectrum.03796-25 (PMC13340073; doi:10.1128/spectrum.03796-25)
Supplement: Supplemental material — Fig. S1 and S2; Tables S1 and S2. [file spectrum.03796-25-s0001.docx]

**Supplementary Materials**

**Title:** Development of a novel, urine-based high-risk Human Papilloma Virus polymerase chain reaction test to predict cervical intraepithelial neoplasia abnormalities associated with cervical cancer

Vasu Saini^1^, Zhengyang Guo^1^, Jinwen Yu^1^, Di Wu^2^, Hui Du^2^, Peng Yin^1^, Garrett Lee Mosley^1^, Wenkui Dai^2^, Ricky Yin To Chiu^1^

^1^ Phase Scientific International Ltd., Hong Kong

^2^ Peking University Shenzhen Hospital, Shenzhen, China

**Supplementary Materials**

**Figure S1:** ROC curve analysis for evaluating the impact of varying the Ct cut-off values for the HPV16 and HPV18 channel to distinguish CIN2+ from <CIN2 cases.

**
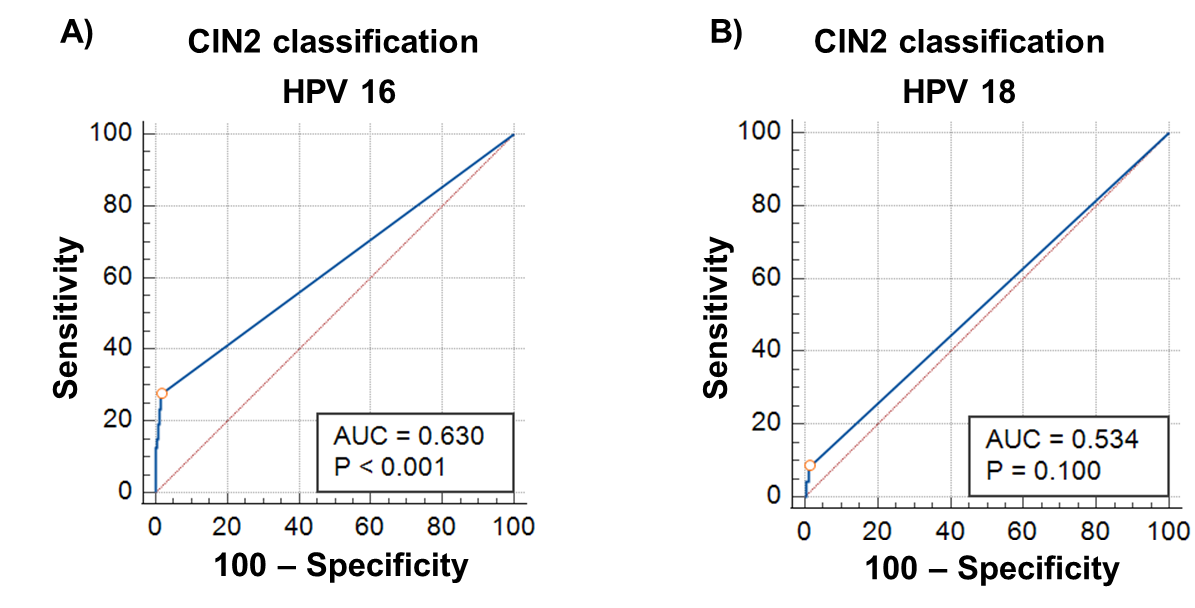
**

**Figure S2:** PR-AUC curve analysis for the 12 other hrHPV, HPV16 and HPV18 channel to distinguish CIN2+ from <CIN2 cases.

**
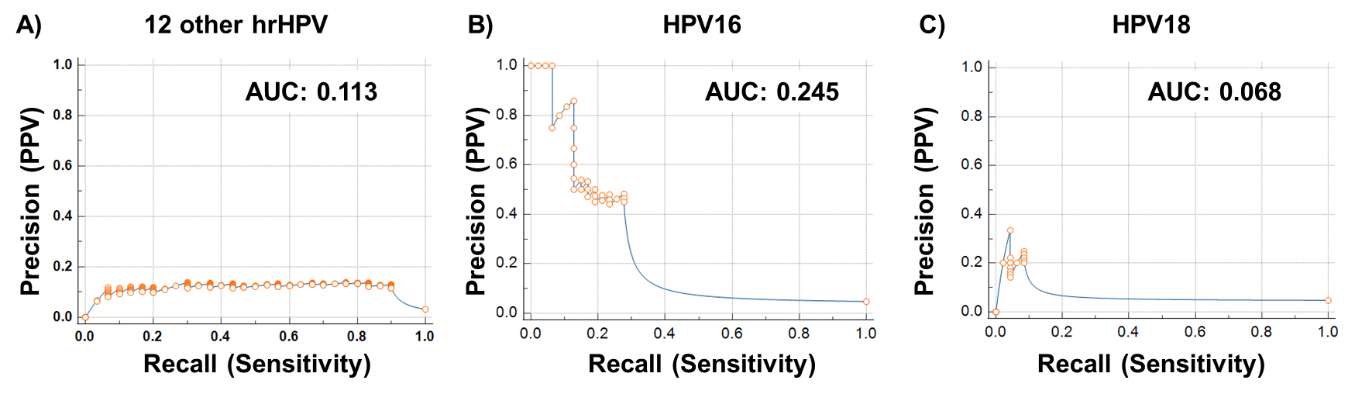
**

**Table S1:** hrHPV positivity and colposcopy results according to age group and total study population in the primary screening and enriched population (Cohort 2).

| **Age group**  **(year)** | **No. of**  **participants** | **No. of participants hrHPV positive results** | | **No. of participants**  **with diseased outcome** | | |
| --- | --- | --- | --- | --- | --- | --- |
|  |  | **Ct cut-off A**  **(HPV16: 38.0, HPV18: 38.0, HPV12+:33.0)** | **Ct cut-off B**  **(HPV16: 38.0, HPV18: 38.0, HPV12+:30.5)** | **<CIN2** | **CIN2+** | **CIN3+** |
| **<30** | 1 | 0/1  (0.00%) | 0/1  (0.00%) | 1/1  (100.0%) | 0/1  (0.00%) | 0/1  (0.00%) |
| **30-39** | 430 | 130/430  (30.23%) | 116/430  (26.98%) | 415/430  (96.51%) | 15/430  (3.49%) | 3/430  (0.69%) |
| **40-49** | 356 | 76/356  (21.35%) | 69/356  (19.38%) | 337/356  (94.66%) | 19/356  (5.34%) | 3/356  (0.84%) |
| **50-59** | 185 | 46/185  (24.86%) | 38/185  (20.54%) | 176/185  (95.14%) | 9/185  (4.86%) | 4/185  (2.16%) |
| **60-65** | 9 | 7/9  (77.78%) | 7/9  (77.78%) | 5/9  (55.56%) | 4/9  (44.44%) | 1/9  (11.11%) |
| **Unknown** | 2 | 0/2  (0.00%) | 0/2  (0.00%) | 2/2  (100.0%) | 0/2  (0.00%) | 0/2  (0.00%) |
| **Total** | 983 | 259/983  (31.44%) | 230/983  23.44% | 936/983  95.22% | 47/983  4.78% | 11/983  1.12% |

**Table S2:** Prevalence of CIN2+ cases in cohort 2 for different hrHPV subtype channels i.e., HPV16, HPV18, 12-other hrHPV, and overall hrHPV at different selected Ct cut-offs.

| **Ct cut-off** | **Prevalence** |
| --- | --- |
|  | **Colposcopy results CIN2+** |
| **HPV16 (Ct cut-off: 38.0)** | 44.83%  (13/29) |
| **HPV18 (Ct cut-off: 38.0)** | 21.05%  (4/19) |
| **12 other hrHPV (Ct cut-off: 33.0)** | 15.06%  (36/239) |
| **12 other hrHPV (Ct cut-off: 30.5)** | 15.69%  (32/204) |
| **hrHPV 12+2 (Ct cut-off A)** | 16.99%  (44/259) |
| **hrHPV 12+2 (Ct cut-off B)** | 18.26%  (42/230) |
